# How Closures Shape Red Wine Characteristics for Medium-Term Storage: Contributions to Explain Orthonasal and Retronasal Perception

**DOI:** 10.3390/foods15101812

**Published:** 2026-05-20

**Authors:** João Mota, Adriana C. S. Pais, Sónia A. O. Santos, Armando J. D. Silvestre, José Pedro Machado, Sílvia M. Rocha

**Affiliations:** 1 LAQV-REQUIMTE, Department of Chemistry, University of Aveiro, Campus Universitário Santiago, 3810-193 Aveiro, Portugal; joao.mota21@ua.pt; 2 CICECO-Aveiro Institute of Materials, Department of Chemistry, University of Aveiro, Campus Universitário Santiago, 3810-193 Aveiro, Portugal; a.c.p.s@ua.pt (A.C.S.P.); santos.sonia@ua.pt (S.A.O.S.); armsil@ua.pt (A.J.D.S.); 3 M.A.Silva Cortiças S.A., Rua Central das Regadas N°49, 4535-167 Mozelos, Portugal; jpmachado@masilva.pt

**Keywords:** wine closures, red wine storage, orthonasal and retronasal perception, chromatography, phenolic compounds, volatile compounds

## Abstract

The sensory perception of wine arises from dynamic interactions processed through two complementary pathways: orthonasal, via inhalation through the nasal cavity, and retronasal, occurring during consumption as compounds are released in the oral cavity and integrated with taste and tactile sensations. Although closures are known to affect oxidative–reductive balance during bottle storage, their impact on orthonasal and retronasal perception remains largely unexplored. To address this gap, a blend red wine from Burgenland, Austria, sealed with Natural Cork, Microagglomerated cork, and Screw Cap closures, was evaluated after 30 months of in-bottle storage. Analyses of physicochemical parameters and phenolic and volatile composition were combined with oxireduction sensory evaluation by a trained panel. Wine sealed with Natural Cork presented a more balanced and complex aroma profile, while wine sealed with a Screw Cap promoted reductive defaults associated with sulfur compounds. Wine sealed with Microagglomerated cork exhibited an intermediate behavior, with phenolic composition positioned between that with Natural Cork and a Screw Cap, and volatile profile showing, with the exception of sulfur compounds, a greater similarity to that with a Screw Cap. Notably, ortho- and retronasal oxireductive scores co-varied, driven by multiple volatile families, and are aligned with oxidation and/or reduction volatile marker content. These findings highlight the role of closure type in modulating both chemical composition and sensory perception, demonstrating that wine–closure pairing can be strategically used to guide the evolution of a wine’s identity during medium-term storage.

## 1. Introduction

Wine aroma is a key driver of quality perception and consumer preference, and it is highly susceptible to post-bottling evolution [1]. Storage-induced changes affect not only aroma profile, intensity, and balance but also taste, mouthfeel, color, and overall chemical composition, ultimately shaping sensory experience [2]. After bottling, wine closures may influence the bottle’s internal environment, modulating these characteristics over time. Different closure systems, namely, Natural Cork, technical closures, and Screw Caps, exhibit distinct barrier properties and oxygen transfer profiles, leading to divergent trajectories of volatile and non-volatile compounds. Natural and technical closures typically allow for moderate oxygen transfer (OTR, 2–4 mg O_2_ per year), whereas Screw Caps have lower OTR, often below 1 mg O_2_ per year. These differences in oxygen ingress influence the wine’s oxidative–reductive balance, triggering chemical and biochemical processes that shape wine quality, stability, and sensory perception [3,4,5,6]. Moderate oxygen ingress can support desirable wine evolution, contributing to color stability and balanced sensory perception, whereas excessive or very low oxygen exposure can induce chemical imbalance, leading to sensory deterioration and the development of oxidative or reductive negative notes. These redox-driven changes affect multiple molecular families, highlighting the broad impact of closure-dependent oxygen transfer on wine overall composition and aroma in particular [7,8,9].

Prior studies have investigated the influence of closure type on wine evolution during bottle storage, reporting effects on physicochemical composition and sensory perception that depend on wine matrix characteristics and storage duration [2,3,4,10,11]. In our previous work [8], two different red wines sealed with distinct closure systems were monitored over short and medium storage periods (5 and 35 months). Closure type modulated wine evolution, with more pronounced differences in volatile composition than in sensory attributes at early stages. However, each wine was assessed under different conditions, so the effects observed could reflect both closure type and wine- or storage time-related changes. The observed discrepancy between ortho- and retronasal perception further highlights the complexity of interpreting closure-related effects.

To understand how closure-dependent changes in wine composition may influence sensory outcomes, it is important to distinguish between orthonasal and retronasal olfaction, which differ in delivery route, physiological context, and sensory experience [12,13]. Orthonasal olfaction refers to the perception of odorants entering the nasal cavity from the external environment during sniffing or normal nasal breathing. Odorant-containing air flows from the nostrils to the olfactory epithelium, generating temporal activation patterns specific to sniff-driven inhalation. Retronasal olfaction, on the other hand, corresponds to the perception of odorants reaching the olfactory epithelium via the oropharynx and nasopharynx, during chewing, swallowing, or exhalation with the mouth closed, and is typically experienced as flavor [14,15,16,17]. In retronasal perception, odorants are often dissolved in saliva, where they may undergo enzymatic transformation [18,19], leading to neural activation distinct from that generated during orthonasal sniffing [17]. Additionally, the distribution of odorants over the olfactory epithelium can differ even for the same molecule, due to differences in airflow and delivery route [20]. While orthonasal perception is primarily determined by headspace composition and sniffing, retronasal perception is shaped by oral processing, including ethanol evaporation, saliva dilution, and mechanical manipulation, creating a complex and time-dependent sensory pattern that cannot be captured by sniffing alone [21,22].

Although it is known that wine closures may affect the oxidative–reductive balance during bottle storage, the temporal impact of different closure types on overall sensory perception remains largely unexplored. To address this gap, the present study investigated how different closure types (Natural Cork, Microagglomerated cork, and Screw Cap), commonly used in wine bottling, modulate the evolution of a red wine over a medium storage period. Instrumental and sensorial data were combined to extract insights into the relationships between physicochemical composition and sensorially detected changes, namely for orthonasal and retronasal perception levels. An Austrian red wine was selected and stored under controlled conditions over 30 months, representing approximately the typical consumption period for red wines [9,23,24]. A holistic, data-driven approach was applied, combining trained-panel sensory evaluation; volatile composition determined with comprehensive volatile profiling by comprehensive gas chromatography-mass spectrometry with a time-of-flight analyzer (GC×GC–ToFMS); phenolic profiling evaluated using ultra-high-performance liquid chromatography, coupled with tandem mass spectrometry (UHPLC–DAD–MS^n^); and general physicochemical analyses.

## 2. Materials and Methods

### 2.1. Materials and Reagents

Cyanidin chloride (>98% purity), gallic acid (>97%), quercetin (>98%), Methyl red indicator, phosphoric acid (>85%), and hydrogen peroxide (35%) were obtained from Sigma Chemical Co. (Madrid, Spain). 3-Octanol (99% purity) was supplied by Aldrich Chemical Company (Milwaukee, WI, USA). Caffeic acid (>95% purity) was purchased from Aldrich Chemicals Co. (Madrid, Spain). Formic acid (96%), catechin (>96%), and ellagic acid (96%) were sourced from Fluka Chemie (Madrid, Spain). HPLC-grade methanol (99.8%) and acetonitrile (99.9%) were acquired from Fisher Scientific Chemicals (Loures, Portugal), while LC–MS-grade water was supplied by Sigma-Aldrich Canada Co. (Oakville, ON, Canada). Sodium chloride (99.8%) was obtained from José Manuel Gomes dos Santos, LDA (Odivelas, Portugal). NaOH (98%) was purchased from Honeywell Specialty Chemicals Seelze GmbH (Seelze, Germany). D-Glucose/D-Fructose Assay Kit was purchased from Megazyme (Bray, Ireland). The retention index standard, consisting of a homologous series of straight-chain n-alkanes (C_8_–C_20_ in hexane), was purchased from Fluka (Buchs, Switzerland). Solid-phase microextraction (SPME) devices for manual sampling, including the fiber assembly, were obtained from Supelco (Aldrich, Bellefonte, PA, USA). The SPME fiber consisted of a 1 cm StableFlex™ (Aldrich, Bellefonte, PA, USA) fused silica fiber coated with divinylbenzene/carboxen/poly(dimethylsiloxane) (DVB/CAR/PDMS, 50/30 µm). Prior to use, the fibers were conditioned following the manufacturer’s instructions.

### 2.2. Red Wine and Closure Samples

A commercial red wine produced from a blend of four red grape varieties (cultivar Blaufränkisch, Cabernet Sauvignon, Merlot, and Zweigelt), originating from the Burgenland region (Mittelburgenland, Austria), was selected for use in this study, which was obtained from a single batch. Red wine samples were sealed using three different closure types: a Natural Cork (Natural Cork), a Microagglomerated cork closure (Micro), and a Screw Cap fitted with a tin–saran liner (Screw Cap). The Natural Cork closure (49 × 24 mm) received a surface treatment consisting of a paraffin emulsion combined with a silicone elastomer blend. The microagglomerated closure (Micro-49 × 24 mm) was produced from cork granules treated with a silicone elastomer blend. The Screw Cap closure (60 × 30 mm) comprised a tin-based metallic layer combined with a poly(vinylidene chloride) (PVDC) liner. The bottles were stored for 30 months before analysis under controlled cellar conditions. Storage was carried out in a horizontal position, protected from light, at temperatures between 14 and 16 °C.

### 2.3. Determination of General Physicochemical Properties of Wine

Analyses were performed on three independent aliquots per bottle, using three bottles per closure type, yielding a total of nine replicates per type of wine bottled with each type of closure (3 bottles × 3 replicates, *n* = 9).

#### 2.3.1. pH

The pH of the wine samples was determined using a calibrated pH meter (micro pH 2002, Crison, Barcelona, Spain). Calibration was performed prior to analysis using standard buffer solutions at pH 4.0 and 7.0.

#### 2.3.2. Alcohol Content

The alcohol content of the wine samples was determined according to the OIV-MA-AS312-01 method [25], based on distillation and a digital densimeter. A 100 mL wine sample was distilled after the addition of a small amount of 2 M calcium hydroxide to prevent acid volatilization. Approximately 80 mL of distillate was collected, and density was measured using a digital densimeter at 20 °C, which converted the density into alcohol content (% *v*/*v*).

#### 2.3.3. Free and Total SO_2_

Free and total sulfur dioxide contents in wine were determined in accordance with the official OIV methods OIV-MA-AS323-04A1 and OIV-MA-AS323-04A2, respectively [25]. For the determination of free sulfur dioxide, approximately 50 mL of wine was placed in a flask and acidified with 15 mL of diluted phosphoric acid to promote sulfur dioxide release. An air or nitrogen stream was passed through the sample, entraining free sulfur dioxide, which was then quantitatively oxidized to sulfuric acid in a hydrogen peroxide solution. The resulting sulfuric acid was titrated with a standardized 0.01 M sodium hydroxide solution (titrant) until the endpoint was reached, using Methyl red as an indicator. The volume of titrant consumed was used to calculate the free sulfur dioxide concentration, expressed as mg/L. Total sulfur dioxide, corresponding to the sum of free and bound forms, was quantified following a similar procedure. Briefly, about 50 mL of wine was acidified with 15 mL of phosphoric acid, and an air stream was applied to transfer sulfur dioxide through a bubbler containing hydrogen peroxide, where it was converted into sulfuric acid. The sulfuric acid generated was then titrated with a standardized 0.01 M sodium hydroxide as described above, and the volume of titrant used allowed for calculation of total sulfur dioxide levels, expressed in mg/L.

#### 2.3.4. Volatile Acidity Deduced from SO_2_

Volatile acidity in red wine was determined by steam distillation followed by titration, in accordance with the OIV-MA-AS313-02 method [25]. Before distillation, dissolved carbon dioxide was removed by vacuum degassing of approximately 50 mL of wine. An aliquot of about 20 mL of the degassed sample was subsequently distilled in the presence of approximately 0.5 g of tartaric acid to facilitate the release of volatile acids. The distillate was titrated with a standardized 0.1 M sodium hydroxide solution using phenolphthalein as the endpoint indicator. The volume of titrant consumed was recorded, and the volatile acidity was expressed as grams of acetic acid per liter (g/L). To account for the contribution of sulfur dioxide, volatile acidity was corrected by subtracting the acidity attributable to free and combined SO_2_.

#### 2.3.5. Total Sugar Content

The content of total sugars (glucose and fructose) in the wine was determined according to the OIV-MA-AS311-02 method [25], using an enzymatic spectrophotometric assay. Glucose and fructose were phosphorylated by adenosine triphosphate (ATP) and converted to glucose-6-phosphate and fructose-6-phosphate, which were subsequently converted into products that reduce nicotinamide adenine dinucleotide phosphate (NADP^+^) to its reduced form (NADPH). The amount of NADPH produced, proportional to the concentration of glucose and fructose, was measured by absorbance at 340 nm, and concentrations were calculated according to the OIV method. Results are expressed as g/L (sum of glucose + fructose).

#### 2.3.6. Chromatic Parameters

The chromatic characteristics of the wine were assessed by spectrophotometry using an Eon microplate reader (BioTek Instruments, Inc., Winooski, VT, USA). Absorbance readings were obtained at 420, 520, and 620 nm. Color intensity (I) was calculated as the sum of absorbance values at these three wavelengths, while shade (N) was expressed as the ratio between absorbance at 420 nm and 520 nm, in accordance with the OIV-MA-AS2-07B method [25]. Furthermore, the relative contributions of the yellow, red, and blue components were determined from the absorbance values at 420, 520, and 620 nm, respectively, according to the method established by Glories [26].

### 2.4. Sensorial Analysis of Red Wine

The sensory evaluation was conducted by a trained panel comprising 6 male assessors from a wine company, all of whom were experienced wine professionals routinely involved in wine sensory evaluation. The panel was trained to evaluate oxidation and reduction level using model solutions and a four-point scale, where 1 corresponded to the wine with the highest reduction and 4 the wine with the highest oxidation level. This scale is routinely used by the company as a standardized approach for the assessment of wine oxidation/reduction status. Continuous training was provided to ensure panel consistency and reliability, with particular emphasis on the sensory recognition of oxidation and reduction. Panel performance was monitored using PanelCheck^®^ software (version 1.4.2, NOFIMA, Tromsø, Norway), which included statistical tools such as Tucker plots and analysis of variance (ANOVA). Sensory sessions were conducted using “XL5-type” wine glasses [27], each containing 30 mL of wine and covered with a watch glass lid. Orthonasal and retronasal evaluations were conducted within the same tasting session for each sample, following a randomized Latin square design. Panelists first evaluated odor perception (orthonasal assessment), followed by flavor perception after tasting (retronasal assessment). The sensory analysis was performed in duplicate over two independent sessions, using one bottle per closure type in each session.

### 2.5. Phenolic Profile of Wine

The methodology applied followed the protocol described in the literature [28], based on the characterization of the phenolic profile using UHPLC–UV–MS^n^ (Thermo Fisher Scientific, San Jose, CA, USA). Briefly, a 1 mL wine sample was filtered through a 0.2 µm PTFE membrane filter before analysis. Injection volumes of 15 µL were introduced into a UHPLC system comprising an UltiMate 3000 LC pump (Thermo Fisher Scientific, San Jose, CA, USA), an UltiMate autosampler (Thermo Fisher Scientific, San Jose, CA, USA) thermostated at 16 °C, and an UltiMate photodiode array detector (80 Hz DAD) (Thermo Fisher Scientific, San Jose, CA, USA). Chromatographic separation was achieved using a Hypersil GOLD RP-C18 column (100 × 2.1 mm, 1.9 µm particle size), preceded by a C18 pre-column (2.1 mm I.D.), both supplied by Thermo Fisher Scientific, and maintained at 40 °C. The mobile phase consisted of solvent A—water (99:1, *v*/*v*) containing 0.1% (*v*/*v*) formic acid—and solvent B: acetonitrile containing 0.1% (*v*/*v*) formic acid. Elution was carried out at a flow rate of 0.30 mL min^−1^ for 22 min, as follows: 99% A from 1 to 2 min; 99–92% A from 2 to 4.5 min; 92–78% A from 4.5 to 14 min; 78–58% A from 14 to 18 min; 58–0% A from 18 to 22 min; 0–99% A from 22 to 26 min, and kept at 0% A (100% B) from 26 to 30 min. UV–Vis chromatograms were recorded at 280, 320, 360, and 520 nm, and molecular absorption spectra were collected over the 210–600 nm range. The UHPLC system was coupled to an LCQ Fleet ion trap mass spectrometer (Thermo Finnigan, San Jose, CA, USA) equipped with an electrospray ionization (ESI) source (Thermo Finnigan, San Jose, CA, USA). The ESI-MS source was operated at a spray voltage of 5 kV, with a capillary temperature of 315 °C. Nitrogen was used as both sheath and auxiliary gas at flow rates of 40 and 5 arbitrary units, respectively. Capillary voltages were set to −35 V in negative ion mode and 47 V in positive ion mode, with tube lens voltages set at −125 V and 115 V, respectively. Collision-induced dissociation (CID) MS^n^ experiments were performed on selected precursor ions over an *m*/*z* range of 100–2000, using an isolation width of 1.0 mass units, a scan time of 100 ms, and a collision energy of 35 arbitrary units. Helium was employed as the collision gas. Data acquisition and processing were carried out using the Xcalibur^®^ software package (version 4.0.27.19, Thermo Finnigan, San Jose, CA, USA). Quantification of phenolic compounds was based on peak areas measured at their respective maximum absorption wavelengths, using external calibration curves prepared from selected reference standards (Table 1). Results were expressed as equivalents of the corresponding standard. All analyses were performed using triplicate injections per bottle (3 bottles × 3 replicates, *n* = 9).

### 2.6. Volatile Profile of Wine

The volatile composition was analyzed by headspace solid-phase microextraction (HS-SPME) combined with GC×GC–ToFMS, following an adaptation of the methodology described in the literature [8]. In brief, 2 mL of wine, 25 μL of the internal standard 3-octanol (810.81 μg/L), and 0.6 g of sodium chloride were placed in a 12 mL vial, at 40.0 ± 0.1 °C for 5 min. Then, the SPME fiber (50/30 µm fused silica fiber coated with divinylbenzene/carboxen/polydimethylsiloxane-DVB/CAR/PDMS) was inserted into the headspace for 10 min while continuously stirring the sample at 250 rpm. After extraction, the SPME fiber was manually introduced into the GC×GC-ToFMS injector at 250 °C. A 0.75 mm I.D. glass liner was used, and splitless injection mode was used (30 s). The system used was a LECO Pegasus BT GC×GC-ToFMS (LECO, St. Joseph, MI, USA), consisting of an Agilent 8890 Gas Chromatograph (Agilent Technologies, Inc., Wilmington, DE, USA) equipped with a dual-stage jet cryogenic modulator (licensed by Zoex) and a secondary oven. A DB-FFAP column (30 m × 0.25 mm I.D., 0.25 μm film thickness; J&W Scientific Inc., Folsom, CA, USA) was used in the first dimension (^1^D), and an Equity-5 column (0.79 m × 0.25 mm I.D., 0.25 μm film thickness; Supelco, Inc., Bellefonte, PA, USA) in the second dimension (^2^D). The carrier gas was helium, flowing at a constant rate of 1.20 mL min^−1^. The oven temperature program began at 30 °C for 2 min, increased to 150 °C for 2 min at 3 °C min^−1^, and then increased to 230 °C for 2 min at 20 °C min^−1^. The secondary oven was set at 5 °C higher than the primary oven. The MS transfer line and source temperatures were both maintained at 250 °C. The modulation period was 4 s, with a modulator offset 15 °C above the secondary oven and modulation pulses alternating between 0.80 s (hot) and 1.20 s (cold). The ToFMS was operated with a spectrum storage rate of 100 spectra per second. The mass spectrometer was set to electron ionization mode (70 eV), scanning an *m*/*z* range of 35–350.

Automated data processing software ChromaTOF^®^ (V5.55.41, LECO, St. Joseph, MI, USA) was used to process total ion chromatograms at a signal-to-noise threshold of 100. The data obtained were transferred into Guineu software (version 0.9, the software source code is published under the GNU General Public License and can be downloaded from the internet https://code.google.com/p/guineu/ (accessed on 10 January 2026)). This software performs score alignment [29] based on first-dimension retention time (^1^*t*_R_), second-dimension retention time (^2^*t*_R_), linear retention index (RI) value, and mass spectrum. For identification purposes, the mass spectra and retention times of each analyte were compared with existing standards, when available, and with values existing in mass spectral libraries, including an in-house library of standards and two commercial databases (Wiley 275 and US National Institute of Science and Technology (NIST) V.2.0–Mainlib and Replib). A mass spectral match factor, similarity >700/1000, was used to decide whether a peak was correctly identified. Moreover, a manual analysis was performed using additional information such as RI value, experimentally obtained through the van Den Dool and Kratz RI equation [30]. To compute the RI, a C_8_-C_20_ n-alkane series was used (solvent n-hexane was used as the C_6_ standard), and then a comparison was performed between these values and those reported in the literature for chromatographic columns equivalent to the first-dimension column (Supplementary Table S1). A 5% difference limit was used when comparing the calculated linear retention index (RI_calc_) with literature data (RI_lit_). The DTIC (deconvoluted total ion current) GC×GC peak area data of the analytes and internal standard (3-octanol) were obtained, and the concentration of each analyte was expressed as μg/L of 3-octanol equivalents. Three independent aliquots of each wine were analyzed per bottle, with a total of 3 bottles per type of closure (3 bottles × 3 replicates, *n* = 9).

### 2.7. Statistical Analysis

Univariate analysis was employed to identify potential significant differences between variables across the conditions studied, within physicochemical, phenolic, and volatile datasets. The statistical tests used included multiple *t*-tests, and One-Way ANOVA (followed by Fisher’s least significant difference test) was applied to evaluate differences in individual physicochemical parameters among the wine closure conditions. Two-Way ANOVA (followed by Tukey’s multiple comparison test) was used for the phenolic and volatile profile datasets. These analyses were performed using GraphPad Prism 8.0.1, with a significance threshold of *p* < 0.05. For the sensory data, the Friedman test was applied using XLSTAT 2023 (Addinsoft, Paris, France), with multiple pairwise comparisons performed using Nemenyi’s post hoc test at a significance level of 5%. Additionally, hierarchical clustering analysis (agglomerative) was conducted on the volatile compounds and combined all the domains of data (sensory analysis, physicochemical parameters, phenolic compounds, and volatile organic compounds), using Euclidean distance as the similarity measure. A Pearson’s correlation matrix was constructed to explore relationships among selected chemical data and sensory evaluation, using MetaboAnalyst 6.0 (web-based software from The Metabolomics Innovation Centre (TMIC), Edmonton, AB, Canada). Before the multivariate analyses, the dataset was normalized by autoscaling.

## 3. Results and Discussion

### 3.1. General Physicochemical Parameters

The general physicochemical characteristics of the Austrian red wine bottled with Natural Cork, Microagglomerated cork (Micro), and Screw Cap closures are summarized in Table 2, which includes the results about pH, free and total sulfur dioxide (SO_2_), volatile acidity deduced from SO_2_, total sugars (glucose and fructose), and alcohol content. These variables represent key oenological indicators for the characterization of wine composition and stability. Table 2 also presents the chromatic parameters, including color intensity, shade, and percentage of yellow, red, and blue.

According to Table 2, no significant differences (*p* > 0.05) were obtained for most general physicochemical parameters under study, with the exception of volatile acidity (F_(2, 24)_ = 20.75, *p* < 0.0001), which exhibits a slightly higher value for the wine bottled with Natural Cork. Given the objective of the present study, particular attention was devoted to the wine oxidation index, based on total SO_2_ measurements, which represent both free and bound forms of SO_2_ [31]. Although no significant differences were observed, the free and total SO_2_ levels tend to be slightly lower in wine bottled with Natural Cork, which may indicate a higher tendency toward oxidation in these bottles.

Analysis of chromatic parameters revealed subtle but significant differences (*p* < 0.05) only in color intensity (F_(2, 24)_ = 20.75, *p* < 0.0001), where wine bottled with Micro and Screw Cap was associated with slightly lower values (8.731 ± 0.012 and 8.564 ± 0.165, respectively). While the present study did not measure OTR, the literature reports that a Screw Cap allows very low oxygen transmission, whereas natural and technical cork allow moderate oxygen ingress [3,4,6,8]. The slight differences observed in general physicochemical parameters may be associated with minor variations in oxygen exposure between closure systems. Nevertheless, as no significant differences were found for most physicochemical and chromatic parameters, the overall impact of this effect appears limited.

### 3.2. Phenolic Profile of Austrian Red Wine

The phenolic compounds identified in the Austrian red wine from the Burgenland region, as well as their retention times, the corresponding [M-H]^−^ and (M^+^) ions (for anthocyanins), and the MS^n^ product ions relevant for their putative identification, are listed in Table 3. A set of 31 phenolic compounds was detected in all the samples; from these 24 exhibited statistically significant differences (*p* < 0.05) among wines sealed with at least two different closures. Wine bottled with a Screw Cap exhibits the most distinct phenolic profile, showing statistically significant differences in 17 compounds compared to wine sealed with Natural Cork and 18 compounds compared to wine sealed with a Micro closure. However, it is important to note that even for compounds exhibiting statistically significant differences (*p* < 0.05), only slight quantitative variations were observed among wines sealed with the three closure types.

Regarding phenolic acids, particular attention was given to those reported as major cork components, namely ellagic and gallic acids, as well as other phenolic compounds described in cork at lower contents, such as caffeic, protocatechuic, and *p*-coumaric acids [32,33]. Among these, ellagic acid showed higher concentrations in the wine sealed with Natural Cork (9.31 ± 0.44 mg/L), followed by Micro (7.99 ± 0.56 mg/L), and lower levels in that with a Screw Cap (7.03 ± 0.38 mg/L), supporting its role as a potential marker of cork-derived phenolic compound migration [34,35]. A similar trend was observed for gallic acid, which also presented higher levels in the wine sealed with Natural Cork and Micro, further suggesting a contribution from the closure material. For the remaining phenolic acids, protocatechuic and caffeic acids also exhibited statistically significant differences, with higher concentrations in wine sealed with Natural Cork, whereas only slight differences were observed for *p*-coumaric acid. Notably, wines sealed with Micro showed a phenolic profile closer to that sealed with Natural Cork, when also compared to wine sealed with a Screw Cap.

**Table 3:** Concentration (mg/L) of phenolic compounds determined by UHPLC-DAD-MS^n^ in Austrian red wine bottled with Natural Cork, Microagglomerated cork (Micro), and Screw Cap closures, and stored for 30 months in a horizontal position. The table includes the retention time (*t*_R_), molecular ion (*m*/*z*), and respective fragmentation data [and references used for identification]. For anthocyanins, the molecular ion is expressed in positive mode.

*t*_R_ (min)	Compound	[M-H]^−^ (*m/z*)	MS^2^ Product Ions (*m*/*z*)		Wine Bottled with (mg/L)		
					Natural Cork	Micro	Screw Cap
***Phenolic acids***							
1.64	Gallic acid ^1^	169	125(100); 81(10)	[36]	90.19 ± 2.84 ^a^	88.15 ± 3.92 ^a^	79.35 ± 3.19 ^b^
2.84	Caftaric acid (stereoisomer) ^2^	311	179(60); 149(100)	[37]	20.91 ± 0.93 ^a^	20.66 ± 0.69 ^a^	13.78 ± 0.33 ^b^
3.11	Protocatechuic acid ^2^	153	123(60); 109(100)	[32]	12.69 ± 0.63 ^a^	10.87 ± 0.55 ^b^	6.64 ± 0.30 ^c^
3.82	Caftaric acid ^2^	311	179(60); 149(100)	[37]	23.87 ± 1.20 ^a^	22.38 ± 0.74 ^a^	19.04 ± 0.70 ^b^
6.17	Coutaric acid ^2^	295	163(100);149(20)	[37]	5.69 ± 0.20 ^a^	5.47 ± 0.24 ^a^	5.25 ± 0.31 ^a^
7.97	Caffeic acid ^2^	179	161(<10); 135(100)	[32]	22.57 ± 0.41 ^a^	19.25 ± 1.31 ^b^	11.50 ± 0.35 ^c^
9.71	*p*-Coumaric acid ^2^	163	119(100)	[32]	29.97 ± 0.63 ^a^	28.40 ± 0.52 ^b^	28.05 ± 0.54 ^b^
12.42	Ellagic acid ^3^	301	257(60); 229(20); 151(80)	[36]	9.31 ± 0.44 ^a^	7.99 ± 0.56 ^b^	7.03 ± 0.38 ^c^
***Flavan-3-ols***							
7.84	Catechin ^4^	289	245(100); 205(40); 179(20)	[37,38]	58.64 ± 2.80 ^a^	48.07 ± 3.32 ^b^	49.05 ± 2.84 ^b^
8.40	Procyanidin type B ^4^	577	451(100); 425(100); 407(40); 289(30)	[38,39]	47.32 ± 3.46 ^a^	54.16 ± 1.33 ^b^	42.58 ± 2.92 ^a^
8.57	Procyanidin type B ^4^	577	451(100); 425(60); 407(40); 289(30)	[38,39]	71.46 ± 5.28 ^a^	68.88 ± 1.70 ^a^	55.51 ± 1.21 ^b^
9.14	Epicatechin ^4^	289	245(100); 205(40); 179(20)	[37,38]	41.06 ± 2.61 ^a^	42.97 ± 1.16 ^a^	42.44 ± 2.53 ^a^
***Flavonols***							
9.46	Kaempferol-glucoside ^5^	447	430(100); 401(70); 355(60); 343(40); 285(60); 244 (40); 179(30)	[37]	0.90 ± 0.05 ^a^	0.78 ± 0.04 ^b^	0.58 ± 0.02 ^c^
11.31	Myricetin-glucuronide ^5^	493	317(100)	[40]	1.93 ± 0.13 ^a^	2.19 ± 0.15 ^ab^	2.21 ± 0.14 ^b^
11.49	Myricetin-3-*O*-galactoside ^5^	479	317(40); 316(100); 271(<10)	[37]	6.38 ± 0.38 ^a^	6.20 ± 0.28 ^a^	5.90 ± 0.32 ^a^
12.67	Quercetin-glucuronide ^5^	477	301(100)	[37,38]	<LOQ	<LOQ	<LOQ
13.10	Laricitrin-3-glucoside ^5^	493	331(100); 330(60)	[41]	2.05 ± 0.15 ^a^	1.60 ± 0.13 ^b^	2.39 ± 0.07 ^c^
14.48	Myricetin ^5^	317	299(<10); 179(100); 151(40)	[38,42]	8.28 ± 0.32 ^a^	7.69 ± 0.30 ^b^	8.18 ± 0.28 ^a^
14.89	Isorhamnetin-hexoside ^5^	477	357(20); 314(100); 285(20)	[43]	0.66 ± 0.02 ^a^	1.05 ± 0.06 ^b^	0.66 ± 0.02 ^a^
15.05	Syringetin-3-glucoside ^5^	507	387(20); 345(40); 344 (100)	[44]	3.01 ± 0.12 ^a^	3.48 ± 0.19 ^b^	2.71 ± 0.20 ^a^
16.93	Quercetin ^5^	301	273(20); 257(29); 179(100); 151(60)	[43]	<LOQ	<LOQ	<LOQ
17.33	Laricitrin ^5^	331	316(100); 179(20); 151(10)	[45]	13.07 ± 0.37 ^a^	12.45 ± 0.93 ^a^	14.60 ± 0.69 ^b^
19.38	Kaempferol ^5^	285	267(60); 257(80); 197(100); 171(80)	[37]	0.88 ± 0.03 ^a^	0.55 ± 0.01 ^b^	0.95 ± 0.02 ^a^
19.75	Isorhamnetin ^5^	315	300(100)	[43]	1.63 ± 0.11 ^a^	1.41 ± 0.03 ^b^	1.61 ± 0.11 ^a^
***Anthocyanins***							
10.76	Peonidin-3-*O*-glucoside ^6^	463	301(100)	[43]	28.47 ± 0.89 ^a^	41.59 ± 1.40 ^b^	45.43 ± 2.51 ^c^
10.96	Malvidin-3-*O*-glucoside ^6^	493	331(100)	[43]	2.40 ± 0.05 ^a^	2.65 ± 0.10 ^b^	2.94 ± 0.06 ^c^
12.43	Delphinidin-3-glucuronide ^6^	479	303(100)	[46]	3.68 ± 0.13 ^a^	4.04 ± 0.15 ^b^	3.48 ± 0.13 ^c^
13.23	Petunidin-3-*O*-glucoside ^6^	479	461(10); 397(10); 317 (100); 221(10)	[43]	<LOQ	<LOQ	<LOQ
14.31	Malvidin-3-*O*-acetylglucoside ^6^	535	383(10); 331(100)	[43]	14.42 ± 0.74 ^a^	14.29 ± 0.41 ^a^	15.64 ± 0.62 ^b^
17.60	Peonidin-3,5-diglucoside ^6^	625	463(100); 421(80)	[43]	2.13 ± 0.04 ^a^	2.38 ± 0.03 ^b^	2.45 ± 0.06 ^b^
18.31	Malvidin-3-*O*-glucoside-4-vinylphenol ^6^	609	447(100)	[47]	<LOQ	<LOQ	<LOQ

*t*_R_—retention time, [M-H]^−^—molecular ion in negative mode, (M^+^)—molecular ion in positive mode. Different lowercase superscripts in a row indicate statistically significant differences between wines bottled with different closures at *p* < 0.05, using multiple *t*-tests and GraphPad Prism. The results are expressed as the averages of 3 bottles × 3 replicates (*n* = 9) ± the standard deviation. ^1^ Expressed in equivalents of gallic acid. ^2^ Expressed in equivalents of caffeic acid. ^3^ Expressed in equivalents of ellagic acid. ^4^ Expressed in equivalents of catechin. ^5^ Expressed in equivalents of quercetin. ^6^ Expressed in equivalents of cyanidin chloride. <LOQ—below limit of quantification.

Among flavan-3-ols, catechin was present in higher amounts in wine sealed with Natural Cork (58.64 ± 2.80 mg/L) than with Micro (48.07 ± 3.32 mg/L) and a Screw Cap (49.05 ± 2.84 mg/L). In general, red wine bottled with Natural Cork tended to exhibit the highest levels of flavan-3-ols, which are known to participate in oxidation reactions during wine storage [48]. Overall, the flavan-3-ol profile follows the same trend for all wines as observed for phenolic acids. The overall flavonol profile remained similar across all wines.

Anthocyanins showed a clear closure-dependent pattern, with lower concentrations generally observed in wine sealed with Natural Cork, intermediate levels in that with Micro closures, and higher concentrations in Screw Cap wines. For instance, peonidin-3-O-glucoside showed concentrations of 28.47 ± 0.89 mg/L, 41.59 ± 1.40 mg/L, and 45.43 ± 2.51 mg/L for wine bottled with Natural Cork, Micro, and a Screw Cap, respectively, while malvidin-3-O-acetylglucoside followed a similar trend. Under oxidative conditions, anthocyanins are known to undergo condensation and polymerization reactions, leading to a decrease in their free forms [49,50]. Overall, these results are consistent with the differences in OTR reported for the different closure systems. The lower oxygen ingress associated with Screw Cap closures may limit oxidative reactions, whereas Microagglomerated cork closures allow moderate oxygen exposure, followed by Natural Cork, promoting post-bottling chemical modifications driven by oxidative processes [8,51,52] and migrations from the closure to the wine [33,44,45,46].

### 3.3. Volatile Profile of Austrian Red Wine

The volatile composition was assessed using HS-SPME/GC×GC-ToFMS, which enables detailed characterization of complex volatile matrices due to its high peak capacity and enhanced separation efficiency compared to one-dimensional GC. The bidimensional separation is achieved according to analytes’ physicochemical properties, primarily polarity in the first dimension and volatility in the second dimension, when a polar/non-polar column configuration is employed (^1^D: DB-FFAP column × ^2^D: Equity-5 column). Such separation is particularly advantageous for wine analysis, where co-elution phenomena frequently occur because of the chemical diversity and wide concentration range of volatile compounds [53].

After pre-data and data processing steps, a set with 168 volatile compounds (Supplementary Material Table S1) was created, which encompassed diverse chemical families, including acids (10), alcohols (26), aldehydes (10), aromatic compounds (2), esters (44), ethers (4), furan derivatives (11), ketones (8), lactones (4), naphthalene compounds (3), norisoprenoids (3), volatile phenols (7), sulfur compounds (5), terpenic compounds (25), and other compounds (6). Among the identified compounds, isoamyl alcohol (^1^*t*_R_ = 678 s; ^2^*t*_R_ = 0.648 s), ethyl hexanoate (^1^*t*_R_ = 738 s; ^2^*t*_R_ = 2.005 s), and ethyl octanoate (^1^*t*_R_ = 1234 s; ^2^*t*_R_ = 2.346 s) were the most abundant. From these 168 analytes, 94 compounds (ca. 56%) showed statistically significant differences among wines sealed with at least two different closures.

**Figure 1:**
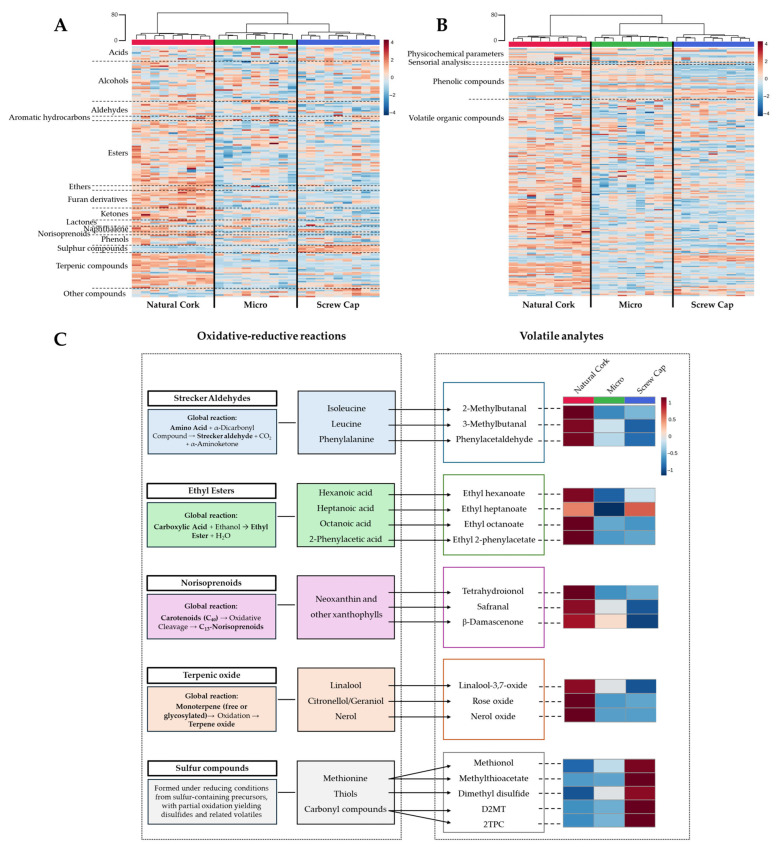
Heatmap, dendrogram, and reactions/analytes associated with oxidative and reductive reactions. (**A**) Heatmap and dendrogram of the 168 volatile compounds identified (Supplementary Table S1), organized by chemical families separated by dashed lines; (**B**) heatmap and dendrogram based on the combined domains of information, including physicochemical parameters, sensory analysis, phenolic compounds, and volatile organic compounds; (**C**) representation of key families associated with the oxidative–reductive status of wine (Strecker aldehydes, ethyl esters, norisoprenoids, terpenic oxides, and sulfur compounds), and their associated volatile compounds (2-methylbutanal, 3-methylbutanal, phenylacetaldehyde, ethyl hexanoate, ethyl heptanoate, ethyl octanoate, β-damascenone, safranal, tetrahydroionol, linalool-3,7-oxide, rose oxide, methionol, methylthioacetate, dimethyl disulfide, dihydro-2-methyl-3(2H)-thiophenone (D2MT), and 2-thiophenecarboxaldehyde (2TPC)), and an indication of the main oxidative and reductive reaction pathways involved. A partial heatmap of these selected compounds, based on mean values for wine sealed with each closure type, is included to illustrate their relative distribution. Euclidean distances are included on the dendrogram *Y*-axis. All heatmaps exhibit a chromatic scale (from dark blue, minimum, to dark red, maximum), and the data from all the domains of information were normalized by autoscaling. Red wine bottled with Natural Cork, Microagglomerated cork (Micro), and Screw Cap closures.

To provide a rapid and visual overview of the volatile pattern of wine sealed with different closures, hierarchical clustering analysis was performed, which allows us to estimate the similarities and differences among the samples under study. The resulting heatmap and dendrogram revealed two main clusters: one corresponding to wine sealed with Natural Cork, and the other comprising wine bottled with Micro and a Screw Cap (Figure 1A). Within this second cluster, a further separation between wine sealed with Micro and a Screw Cap was observed, indicating smaller differences between these closure types. The relative abundance of each compound is represented using a chromatic scale (from dark blue to dark red, the minimum and the maximum, respectively), unveiling that wine sealed with Natural Cork generally exhibited higher amounts of volatile compounds. A similar clustering pattern was obtained (with a similar Euclidean distance—80) when all domains of information were considered (physicochemical parameters, sensory analysis, phenolic composition, and volatile compounds), which also revealed two main clusters, one corresponding to the wine sealed with Natural Cork and the other with wine bottled with Micro and a Screw Cap (Figure 1B). This consistent clustering reinforces the influence of closure type on wine composition, indicating that wine sealed with Natural Cork tends to present slightly lower levels of phenolic compounds and higher concentrations of volatile compounds, namely esters and terpenic compounds that significantly contribute to the fruity and floral-like aromas of wine (Figure 1A). The observed clustering according to closure type reflects a discernible effect of closure on the volatile composition and its impact on the oxidative–reductive balance.

Detailed analysis focused on compounds previously associated with oxidative and reductive reactions, as illustrated in Figure 1C, including Strecker aldehydes, ethyl esters, norisoprenoids, terpenic oxides, and sulfur compounds. Strecker aldehydes, such as 2-methylbutanal, 3-methylbutanal, and phenylacetaldehyde, originate from the oxidative degradation of isoleucine, leucine, and phenylalanine, respectively, while norisoprenoids, including β-damascenone, safranal, and tetrahydroionol, are formed via oxidative cleavage of carotenoids, mainly neoxanthins and other xanthophylls, reflecting oxidative reactions during in-bottle storage conditions. Ethyl esters, namely ethyl hexanoate, ethyl heptanoate, ethyl octanoate, and ethyl 2-phenylacetate, derived from their corresponding acids, contribute to fruity and floral aromas [54] and are influenced by both hydrolytic and oxidative reactions during bottle storage. Terpenic compounds, which contribute floral, fruity, citrus, and herbal notes [55], were represented by three monoterpenic oxides—linalool-3,7-oxide, rose oxide, and nerol oxide, formed from linalool, geraniol, and nerol, respectively—and analyzed as markers of oxidative transformations. In contrast, sulfur-containing compounds, including methionol and methylthioacetate derived from methionine, dimethyl disulfide originating from thiols, and dihydro-2-methyl-3(2H)-thiophenone and 2-thiophenecarboxaldehyde produced from carbonyl-containing sulfur compounds, were analyzed as indicators of reductive processes.

Considering the analytes selected for Figure 1C, it was observed that 3-methylbutanal was present in higher levels in wine bottled with Natural Cork (4.037 ± 0.659 mg/L) than with Micro (3.393 ± 0.439 mg/L) and a Screw Cap (2.986 ± 0.630 mg/L), with a statistically significant difference between Natural Cork and a Screw Cap. A similar trend was observed for β-damascenone, a norisoprenoid contributing floral notes known to mask the perception of certain aromas [56,57], with concentrations of 1.399 ± 0.096 µg/L in Natural Cork, 1.165 ± 0.031 µg/L in Micro, and 0.841 ± 0.054 µg/L in Screw Cap, showing significant differences across the three closure types (*p* < 0.05). Among ethyl esters, ethyl octanoate, associated with sweet and fruity aromas [58], was notably higher in wine bottled with Natural Cork (874.256 ± 77.852 µg/L) compared to Micro (495.923 ± 36.067 µg/L) and a Screw Cap (473.477 ± 39.811 µg/L). Rose oxide, a terpenic oxide with sweet and fruity notes [59], followed the same pattern, reaching 0.406 ± 0.033 µg/L in Natural Cork, whereas its levels were lower in Micro (0.228 ± 0.016 µg/L) and Screw Cap (0.234 ± 0.020 µg/L). In contrast, methionol, indicative of reductive conditions, increased from 5.452 ± 0.345 mg/L in Natural Cork to 6.404 ± 0.903 mg/L in Micro, reaching 8.198 ± 0.816 mg/L in Screw Cap, consistent with reduced oxygen availability promoting sulfur compound accumulation. Collectively, these results indicate that wine bottled with Natural Cork exhibits a moderate oxidative–reductive balance, supporting the expression of floral and fruity aromas, whereas wine bottled with a Screw Cap develops a more reductive environment, giving rise to perceptible reductive notes. Wine sealed with Micro exhibited a volatile profile more similar to that sealed with a Screw Cap, except for the sulfur compounds.

In addition to the oxidative–reductive markers, and as described in the previous section, wine bottled with Natural Cork may receive small amounts of compounds released through cork migration, such as camphor [60]. This bicyclic monoterpene ketone, known for its fresh, minty, and eucalyptus-like aroma [11,61], was exclusively detected in wine bottled with Natural Cork (0.283 ± 0.044 µg/L), consistent with previous studies identifying camphor as a key marker of Natural Cork stoppers [8,11,60,61,62].

### 3.4. Orthonasal and Retronasal Sensory Evaluation of Austrian Red Wine

**Figure 2:**
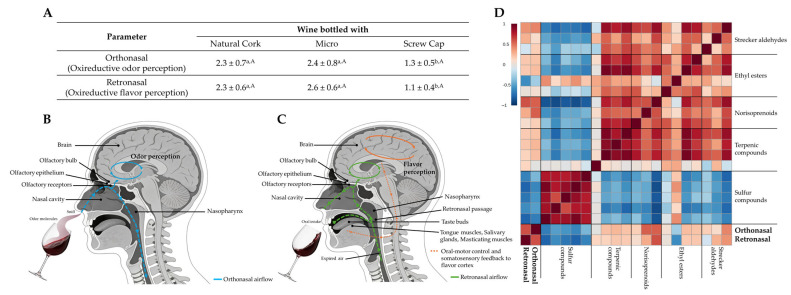
Evaluation of the effect of the closure type on the oxidative–reductive sensory profile (orthonasal and retronasal perception) and its relation with selected volatiles determined in the Burgenland red wine stored for 30 months: (**A**) sensory analysis of oxireduction levels in wine sealed with Natural Cork, Microagglomerated cork (Micro), and a Screw Cap, evaluated on a 1–4 scale (1 = most reductive, 4 = most oxidative). Different superscript lowercase letters in a row indicate statistically significant differences between wines sealed with different closure types (*p* < 0.05, Friedman’s test; *n* = 6 tasters, each wine evaluated twice, averaged ± standard deviation). The same uppercase letter in a column indicates no statistically significant differences between orthonasal and retronasal evaluation of the same wine sealed with the same closure type (*p* > 0.05, Friedman’s test; *n* = 6 tasters, each wine evaluated twice, averaged ± standard deviation). (**B**) Orthonasal pathway illustrating odor perception (orthonasal perception) from the nose to the brain, adapted from [17]; (**C**) retronasal pathway illustrating flavor perception (retronasal perception) from the mouth to the brain, adapted from [17]; (**D**) Pearson’s correlation matrix linking target volatile compounds (Strecker aldehydes, ethyl esters, norisoprenoids, terpenic compounds, and sulfur compounds) with orthonasal and retronasal sensory perception. All variables were autoscaled and represented using a chromatic scale ranging from dark blue (minimum) to dark red (maximum).

To explore how closure type influences wine oxidative and reductive sensorial status, a sensory evaluation was conducted focusing on both orthonasal and retronasal perception, using a four-point scale, where 1 represented the wine with the most reductive character and 4 the wine with the most oxidized character. Orthonasal evaluation captured the oxidative–reductive odor perception, while retronasal evaluation measured the oxidative–reductive flavor perception (Figure 2A).

Statistically significant differences were observed at the orthonasal level between wines sealed with Natural Cork and Micro closures and those bottled with a Screw Cap (Figure 2A). Wine sealed with a Screw Cap exhibited a more pronounced reductive character (1.3 ± 0.5). Similarly, retronasal perception revealed significant differences, particularly between wine sealed with Natural Cork and Micro (2.3 ± 0.6 and 2.6 ± 0.6, respectively) and those with a Screw Cap (1.1 ± 0.4). The observed score for the wine sealed with Natural Cork and Micro (in the middle of the scale, approximately 2) allows us to infer a balanced oxidation status. Across both perception pathways, the relatively high standard deviations observed, particularly within the four-point scale, indicate considerable variability among panelists, reflecting inherent subjectivity and differences in individual sensitivity to wine oxireductive status evaluation. Additionally, the relatively small sensory panel, composed exclusively of male assessors, may represent a limitation of the study regarding the broader extrapolation of the sensory results.

Despite ortho- and retronasal perception differing in delivery route, physiological context, and possible sensory experience, the results obtained indicated that the ortho- and retronasal scores vary in the same direction. Orthonasal perception (Figure 2B) involves the inhalation (“sniffing”) of the wine, in which volatile compounds released from the wine to the headspace enter the nasal cavity and reach the olfactory epithelium, being processed primarily as isolated olfactory signals [16,17]. Odorant receptors are located on olfactory receptor cells in the nasal cavity. Each olfactory receptor cell expresses only one type of odorant receptor, and each receptor can detect a limited number of odorant substances [63]. This process is closely approximated by the advanced chromatographic methodology used (HS-SPME/GC×GC-ToFMS), in which volatile and semi-volatile analytes are released from the wine matrix, partitioned into the headspace, then are sorbed onto the SPME stationary phase [53] and desorbed into the GC×GC injector. Although this chromatographic method provides a detailed and highly sensitive profile of the wine’s volatile composition, some differences detected in the volatile profiles may not be perceived by the human nose. In this case, the oxireductive status of the wine assessed by the trained panel is in accordance with chemical data obtained for the potential markers of the oxidation and reduction reactions (Figure 1C).

The retronasal perception (Figure 2C) occurs during wine consumption, when volatile compounds released in the oral cavity are transported through the retronasal pathway to the nasopharynx and subsequently reach the olfactory epithelium [14,15,16,17]. This process is accompanied by gustatory and somatosensory input from taste buds and saliva, allowing the brain to integrate these cues into a unified flavor percept. Theoretically, some retronasal odorants may be generated only after in-mouth transformations, modulated, for example, by enzymes, such as hydrolysis of the glycosidically linked volatiles. In those cases, they may be undetectable orthonasally. However, regarding the evaluation of the oxidative–reductive sensory profile of wine, both types of analysis provide similar results (Figure 2A). These results allow us to infer that the potential oxidative–reductive volatile markers are already present in both sensorial evaluation routes, i.e., in the wine headspace associated with orthonasal perception and after crossing the oral cavity until the olfactory epithelium is reached. Actually, the spatiotemporal activation patterns across receptor populations can differ for the same odorant orthonasally versus retronasally; the same olfactory receptor neurons and receptor repertoire are engaged by both ortho- and retronasal routes.

To further explore the relationship between wine volatile profile and sensory perception, a Pearson’s correlation matrix, at a 5% confidence level, is visualized in a red-to-blue color scale, with the colors ranging from dark red ρ = +1 to dark blue ρ = −1 (Figure 2D). This matrix was constructed for the target families analyzed in Section 3.3 (Strecker aldehydes, ethyl esters, norisoprenoids, terpenic compounds, including terpenic oxides and camphor, and sulfur compounds—Figure 1C and Table S1) and both orthonasal and retronasal evaluations (Figure 2A). With the exception of the sulfur compounds, most of the targeted compounds (esters, oxide terpenes, norisoprenoids, and Strecker aldehydes) increased with the same trends, albeit with different magnitudes, and they were also positively correlated with the ortho- and retronasal scores (higher score corresponded to higher oxidative notes). On the other hand, sulfur compounds, detectable at low concentrations [64], were correlated with each other, but varied in the opposite direction to analytes related to oxidation reactions and were also negatively correlated with the ortho- and retronasal score (lower score corresponded to higher reductive notes).

## 4. Conclusions

This study provides evidence on how different closure types modulate the physicochemical and sensorial characteristics of red wine over a medium in-bottle storage term, addressing the previously unexplored temporal impact on oxidative–reductive perception. In general, no significant differences (*p* > 0.05) were obtained for the general physicochemical parameters under study, with the exception of volatile acidity, which exhibited a slightly higher value for the wine bottled with Natural Cork. However, slight differences were detectable on the phenolic and volatile profiles, as well as on the sensorial oxireductive status of wines. Differences in the phenolic profiles seem to be mechanistically consistent with the OTR reported for each closure: Screw Caps (low OTR) limit oxidation, preserving free anthocyanins; Natural Cork closures (higher OTR) promote oxidative reactions, reducing free anthocyanins but increasing flavan-3-ol involvement and cork-derived phenolics, such as acids; and Microagglomerated cork closures exhibit an intermediate profile. Regarding the volatile profile, wine sealed with Natural Cork generally maintained higher levels of Strecker aldehydes, ethyl esters, terpenic compounds, and norisoprenoids. This chemical profile resulted in a more balanced and complex sensory profile, with enhanced varietal, fruity, and floral positive notes, which are generally appreciated by consumers. In contrast, wine sealed with a Screw Cap showed higher concentrations of sulfur compounds, associated with reductive off-flavors, leading to a more reductive sensory profile, particularly evident during sensory evaluation. Except for sulfur compounds, wines sealed with Micro closures exhibited a volatile profile closer to that of Screw Cap wines.

For a medium storage term, the ortho- and retronasal scores associated with oxireductive status vary in the same direction and reflect a sensorial perception response involving multiple reactions that occur inside the bottle, modulated by the type of closure. From an enological perspective, these results suggest that closure selection can contribute to shaping a wine’s sensory expression under the studied conditions, which may be relevant for consumers’ overall wine experience.

## Figures and Tables

**Table 1:** Calibration data used for the quantification of phenolic compounds in Austrian red wine.

Compound	Wavelength (nm)	Concentration Range (mg/L)	Calibration Curve ^a^	r^2^	LOD	LOQ
Caffeic acid	280	0.20–102.0	y = 58,012x − 26,782	0.9999	1.72	5.21
Catechin	280	0.42–84.8	y = 7120x + 7717.2	0.9996	2.00	6.06
Cyanidin chloride	520	1.00–60.0	y = 121,121x − 107,068	0.9999	0.64	1.94
Ellagic acid	360	0.31–61.2	y = 38,415x + 9853.8	0.9993	1.87	5.66
Gallic acid	280	0.61–102.0	y = 54,851x − 8249.8	0.9998	2.10	6.37
Quercetin	360	0.10–20.4	y = 106,045x − 5670	0.9995	0.55	1.68

^a^ y = peak area, x = concentration in mg/L, r^2^—correlation coefficient, LOD—limit of detection, LOQ—limit of quantification.

**Table 2:** General physicochemical and chromatic characterization of Austrian red wine bottled with Natural Cork, Microagglomerated cork (Micro), and Screw Cap closures, and stored for 30 months in a horizontal position. The results are expressed as the averages of 3 bottles × 3 replicates (*n* = 9) ± the standard deviation.

Parameter	Wine Bottled with		
	Natural Cork	Micro	Screw Cap
pH	3.72 ± 0.01 ^a^	3.72 ± 0.01 ^a^	3.73 ± 0.01 ^a^
Alcohol content (% vol.)	14.71 ± 0.01 ^a^	14.69 ± 0.02 ^a^	14.70 ± 0.02 ^a^
SO_2_ free (mg SO_2_/L)	13 ± 2 ^a^	14 ± 1 ^a^	14 ± 2 ^a^
SO_2_ total (mg SO_2_/L)	66 ± 5 ^a^	70 ± 5 ^a^	71 ± 3 ^a^
Volatile acidity deduced from SO_2_ (g/L acetic acid)	0.748 ± 0.009 ^a^	0.717 ± 0.016 ^b^	0.726 ± 0.013 ^b^
Total sugar (g/L)	2.8 ± 0.0 ^a^	2.8 ± 0.0 ^a^	2.8 ± 0.1 ^a^
I (Abs420 + Abs520 + Abs620)	9.364 ± 0.272 ^a^	8.731 ± 0.012 ^b^	8.564 ± 0.165 ^b^
N (Abs420/Abs520)	0.979 ± 0.017 ^a^	0.966 ± 0.012 ^a^	0.973 ± 0.012 ^a^
%yellow (Abs420/I × 100)	43.614 ± 0.405 ^a^	43.311 ± 0.295 ^a^	43.464 ± 0.302 ^a^
%red (Abs520/I × 100)	44.538 ± 0.365 ^a^	44.827 ± 0.341 ^a^	44.693 ± 0.252 ^a^
%blue (Abs620/I × 100)	11.847 ± 0.098 ^a^	11.863 ± 0.321 ^a^	11.843 ± 0.056 ^a^

Different superscript lowercase letters in a row represent statistically significant differences between wines sealed with different closures at *p* < 0.05. Statistical significance assessed using One-Way ANOVA (followed by Tukey’s multiple comparison test) in GraphPad Prism.

## Data Availability

The original contributions presented in this study are included in the article/Supplementary Materials. Further inquiries can be directed to the corresponding author.

## Supplementary Materials

The following supporting information can be downloaded at: https://www.mdpi.com/article/10.3390/foods15101812/s1, Table S1: List of the volatile compounds putatively identified in the Austrian red wine sealed with Natural Cork, Microagglomerated (Micro), and Screw Cap closures using HS-SPME/GC×GC-ToFMS. The wines were stored for 30 months, in a horizontal position, at temperatures between 14 and 16 °C. Relevant chromatographic data used to assess compound identification is also included. References [65,66,67,68,69,70,71,72,73,74,75,76,77,78,79,80,81,82,83,84,85,86,87,88,89,90,91,92,93,94,95,96,97,98,99,100,101,102,103,104,105,106,107,108,109,110,111,112,113,114,115,116,117,118,119,120,121,122,123,124,125,126,127,128,129,130,131,132,133,134,135,136,137,138,139,140,141] are cited in the Supplementary Materials.
