# Supplementary material for: How Closures Shape Red Wine Characteristics for Medium-Term Storage: Contributions to Explain Orthonasal and Retronasal Perception
Source: Foods. 2026 May 20;15(10):1812. doi: 10.3390/foods15101812 (PMC13205760; doi:10.3390/foods15101812)
Supplement: Supplementary file 1 [file foods-15-01812-s001.zip › foods-4315649-supplementary.pdf]

Supplementary material

Table S1. List of the volatile compounds putatively identified in the Austrian red wine sealed with Natural Cork, Microagglomerated (Micro), and Screw Cap closures using HS-SPME/GC×GC-ToFMS. The wines were stored for 30 months, in a horizontal position, at temperatures between 14 and 16 °C. Relevant chromatographic data used to assess compound identification is also included.

$^{1}t_{R}$ (s)[#]	$^{2}t_{R}$ (s)[#]	Volatile compounds	CAS	RI $_{Calc}$[$]	RI $_{Lit}$[*]	Wine bottled with (μg/L)[&]		
						**Natural Cork**	**Micro**	**Screw Cap**
		Acids						
1286	0.418	Acetic acid	64-19-7	1461	1452 [65]	45.625 ± 5.648[a]	45.625 ± 5.648[a]	44.917 ± 7.353[a]
1562	0.454	Isobutyric acid	79-31-2	1575	1554 [66]	7.239 ± 0.253[a]	7.071 ± 0.666[a]	7.640 ± 0.364[a]
1702	0.446	Butyric acid	107-92-6	1635	1636 [65]	21.493 ± 3.020[a]	18.970 ± 4.799[a]	20.482 ± 3.949[a]
1726	0.403	2-Propenoic acid	79-10-7	1646	--	0.120 ± 0.019[a]	0.100 ± 0.019[a]	0.118 ± 0.014[a]
1790	0.473	Isovaleric acid	503-74-2	1675	1666 [65]	14.684 ± 1.843[a]	14.965 ± 2.895[a]	14.506 ± 2.201[a]
1942	0.454	Pentanoic acid	109-52-4	1744	1733 [65]	0.148 ± 0.012[a]	0.173 ± 0.024[a]	0.158 ± 0.017[a]
2170	0.491	Hexanoic acid	142-62-1	1850	1839 [65]	13.906 ± 2.229[a]	11.621 ± 3.147[a]	12.677 ± 1.562[a]
2382	0.494	Heptanoic acid	111-14-8	1929	1946 [66]	0.223 ± 0.032[a]	0.266 ± 0.076[a]	0.259 ± 0.047[a]
2590	0.566	Octanoic acid	124-07-2	2052	2068 [67]	33.434 ± 1.475[a]	31.736 ± 0.835[a]	32.253 ± 1.526[a]
2822	0.423	Decanoic acid	334-48-5	2156	2250 [68]	2.209 ± 0.256[a]	1.587 ± 0.343[a]	2.754 ± 0.195[b]
		Alcohols						
310	0.523	1-Propanol	71-23-8	1042	1045 [69]	8.761 ± 0.457[a]	7.838 ± 1.118[a]	8.752 ± 0.451[a]
438	0.678	3-Pentanol	584-02-1	1120	1111 [70]	0.179 ± 0.009[a]	0.166 ± 0.011[a]	0.177 ± 0.009[a]
514	0.591	1-Butanol	71-36-3	1157	1145 [71]	4.731 ± 0.248[a]	4.405 ± 0.235[b]	4.715 ± 0.266[ab]

550	0.589	1-Peten-3-ol	616-25-1	1186	1166 [72]	1.118 ± 0.415[a]	0.615 ± 0.106[b]	0.769 ± 0.174[ab]
678	0.648	Isoamyl alcohol	123-51-3	1230	1207 [73]	772.687 ± 43.265[a]	548.711 ± 21.882[b]	676.108 ± 37.429[c]
770	0.651	1-Pentanol	71-41-0	1268	1255 [74]	6.329 ± 2.008[a]	7.990 ± 1.481[a]	8.068 ± 1.081[a]
926	0.710	Isohexyl alcohol	626-89-1	1327	1311 [75]	3.617 ± 0.176[a]	3.484 ± 0.180[a]	3.515 ± 0.132[a]
942	0.877	2-Heptanol	543-49-7	1328	1327 [76]	1.260 ± 0.047[a]	1.252 ± 0.049[a]	1.218 ± 0.060[a]
958	0.727	3-Methyl-1-pentanol	589-35-5	1339	1331 [77]	4.711 ± 0.213[a]	4.578 ± 0.128[a]	4.698 ± 0.225[a]
1026	0.732	1-Hexanol	111-27-3	1365	1363 [78]	23.752 ± 4.980[a]	27.991 ± 4.375[a]	20.791 ± 5.705[a]
1098	0.674	3-Hexen-1-ol	928-96-1	1392	1400 [79]	4.403 ± 0.164[a]	4.671 ± 0.180[a]	4.327 ± 0.160[a]
1182	0.650	2-Hexen-1-ol	928-95-0	1416	1407 [72]	0.898 ± 0.031[a]	0.899 ± 0.062[a]	0.860 ± 0.065[a]
1194	0.999	2-Octanol	123-96-6	1425	1412 [80]	0.579 ± 0.019[a]	0.596 ± 0.019[a]	0.561 ± 0.061[a]
1222	0.872	7-Octen-4-ol	53907-72-5	1456	1453 [81]	1.568 ± 0.211[a]	1.680 ± 0.148[a]	1.728 ± 0.186[a]
1278	0.799	1-Heptanol	111-70-6	1459	1456 [82]	6.295 ± 0.296[a]	6.365 ± 0.279[a]	6.220 ± 0.233[a]
1366	0.896	2-Ethyl-1-hexanol	104-76-7	1497	1493 [83]	1.275 ± 0.165[a]	1.224 ± 0.176[a]	1.057 ± 0.146[a]
1378	0.719	4-Hepten-1-ol	20851-55-2	1503	1502 [70]	0.756 ± 0.100[a]	0.800 ± 0.148[a]	0.748 ± 0.053[a]
1514	2.244	4-tert-Butylcyclohexanol	98-52-2	1696	--	1.498 ± 0.265[a]	2.357 ± 0.369[b]	1.352 ± 0.205[a]
1438	1.090	2-Nonanol	628-99-9	1523	1522 [71]	1.383 ± 0.113[ab]	1.322± 0.107[a]	1.468 ± 0.086[b]
1530	0.876	1-Octanol	111-87-5	1564	1561 [71]	4.768 ± 0.312[a]	4.706 ± 0.250[a]	4.950 ± 0.208[a]
1666	0.771	2-Octen-1-ol	18409-17-1	1621	1620 [84]	0.356 ± 0.058[a]	0.454 ± 0.172[a]	0.327 ± 0.099[a]
1770	0.946	1-Nonanol	143-08-8	1666	1668 [85]	2.700 ± 0.275[a]	2.373 ± 0.237[a]	3.134 ± 0.237[b]
1902	1.269	2-Undecanol	1653-30-1	1726	1717 [86]	0.109 ± 0.017[a]	0.071 ± 0.015[b]	0.161 ± 0.019[c]
1982	0.648	Dimethylbenzenemethanol	617-94-7	1763	1776 [87]	0.024 ± 0.007[a]	0.022 ± 0.006[a]	0.017 ± 0.005[a]
2222	0.519	Benzyl Alcohol	100-51-6	1877	1908 [88]	7.889 ± 0.197[a]	7.443 ± 0.407[a]	8.376 ± 0.269[b]
2282	0.578	Benzeneethanol	60-12-8	1905	1920 [65]	334.730 ± 24.372[a]	327.906 ± 20.210[a]	300.253 ± 47.512[a]

		Aldehydes						
94	0.416	Acetaldehyde	75-07-0	700	718 [79]	15.617 ± 1.516[a]	14.035 ± 3.129[a]	14.691 ± 1.527[a]
182	0.701	2-Methylbutanal	96-17-3	927	915 [65]	3.390 ± 0.464[a]	2.012 ± 0.84[b]	2.189 ± 0.954[ab]
186	0.692	3-Methylbutanal	590-86-3	932	922 [65]	4.037 ± 0.659[a]	3.393 ± 0.439[ab]	2.986 ± 0.630[b]
402	1.055	Hexanal	66-25-1	1080	1087 [65]	0.814 ± 0.045[a]	0.743 ± 0.048[b]	0.526 ± 0.053[c]
866	1.602	Octanal	124-13-0	1291	1295 [89]	1.224 ± 0.208[a]	1.486 ± 0.273[b]	0.936 ± 0.161[a]
1126	1.710	Nonanal	124-19-6	1397	1385 [90]	7.401 ± 0.735[a]	6.615 ± 1.536[ab]	5.224 ± 1.284[b]
1270	1.834	Decanal	112-31-2	1500	1499 [91]	6.130 ± 1.162[a]	6.465 ± 1.007[a]	5.409 ± 2.010[a]
1438	0.712	Benzaldehyde	100-52-7	1523	1523 [73]	3.878 ± 0.174[a]	3.878 ± 0.174[a]	4.280 ± 0.416[a]
1718	0.704	Phenylacetaldehyde	122-78-1	1643	1652 [65]	5.466 ± 0.377[a]	4.255 ± 0.442[b]	3.703 ± 0.250[c]
1790	0.681	Hydroxybenzaldehyde	90-02-8	1675	1674 [92]	0.027 ± 0.006[a]	0.025 ± 0.006[a]	0.029 ± 0.008[a]
		Aromatic compounds						
598	1.368	Xylene	108-38-3	1143	1142 [93]	2.035 ± 0.621[a]	1.415 ± 0.248[a]	1.838 ± 0.385[a]
966	1.547	Ethylmethylbenzene	622-96-8	1227	1226 [94]	0.317 ± 0.101[a]	0.140 ± 0.027[a]	0.245 ± 0.106[a]
		Esters						
130	0.523	Methyl acetate	79-20-9	842	825 [95]	7.571 ± 0.637[a]	5.876 ± 1.727[ab]	5.504 ± 1.107[b]
230	1.023	Ethyl propanoate	105-37-3	963	961 [96]	13.872 ± 2.323[a]	11.477 ± 1.719[a]	13.071 ± 2.073[a]
242	1.121	Ethyl isobutyrate	97-62-1	968	967 [97]	59.262 ± 2.318[a]	56.334 ± 3.700[a]	44.025 ± 3.692[a]
250	0.886	Propyl acetate	109-60-4	976	983 [98]	2.494 ± 0.149[a]	2.564 ± 0.189[a]	2.498 ± 0.236[a]
262	0.895	Methyl butanoate	623-42-7	986	989 [99]	0.111 ± 0.005[a]	0.143 ± 0.022[b]	0.118 ± 0.010[a]
298	1.043	Isobutyl acetate	110-19-0	1013	1013 [94]	24.012 ± 3.596[a]	16.044 ± 4.145[b]	16.586 ± 4.427[b]
330	1.175	Ethyl butyrate	105-54-4	1032	1020 [97]	62.802 ± 1.350[a]	58.721 ± 1.199[a]	59.441 ± 1.426[a]
354	1.587	Ethyl 2-methylbutanoate	7452-79-1	1048	1043 [100]	16.206 ± 1.410[a]	15.205 ± 1.279[a]	14.413 ± 1.207[a]

382	1.539	Ethyl 3-methylbutanoate	108-64-5	1066	1064 [100]	36.530 ± 1.169[a]	35.384 ± 2.856[a]	34.199 ± 2.350[a]
390	1.204	Butyl acetate	123-86-4	1072	1075 [72]	0.500 ± 0.041[a]	0.433 ± 0.048[b]	0.478 ± 0.042[ab]
402	1.559	Isobutyl propanoate	540-42-1	1078	1083 [101]	0.069 ± 0.004[a]	0.075 ± 0.015[a]	0.069 ± 0.004[a]
458	0.929	Diethyl carbonate	105-58-8	1110	1083 [102]	0.117 ± 0.018[a]	0.131 ± 0.026[a]	0.074 ± 0.015[b]
506	1.663	Ethyl pentanoate	539-82-2	1133	1134 [103]	1.983 ± 0.089[a]	1.939 ± 0.084[a]	1.973 ± 0.081[a]
574	1.051	Ethyl 2-butenoate	10544-63-5	1167	1165 [83]	0.884 ± 0.018[a]	0.845 ± 0.038[a]	0.871 ± 0.015[a]
594	1.538	Isoamyl acetate	123-92-2	1120	1112 [71]	233.601 ± 6.981[a]	189.466 ± 14.107[b]	186.589 ± 9.766[b]
626	1.556	Methyl hexanoate	106-70-7	1193	1189 [104]	1.906 ± 0.158[ab]	1.727 ± 0.086[b]	1.857 ± 0.061[a]
626	2.039	Isoamyl propanoate	105-68-0	1194	1183 [70]	1.871 ± 0.347[a]	1.488 ± 0.270[b]	1.751 ± 0.324[ab]
738	2.005	Ethyl hexanoate	123-66-0	1247	1244 [103]	317.780 ± 30.756[a]	256.157 ± 40.791[a]	279.979 ± 26.793[a]
834	1.772	Hexyl acetate	142-92-7	1277	1279 [87]	4.589 ± 0.153[a]	4.200 ± 0.182[b]	4.568 ± 0.199[a]
986	2.202	Ethyl heptanoate	106-30-9	1336	1332 [71]	11.536 ± 0.441[ac]	10.267 ± 0.504[b]	11.634 ± 0.568[c]
1030	2.541	Isobutyl hexanoate	105-79-3	1353	1347 [75]	1.520 ± 0.083[ac]	1.347 ± 0.109[b]	1.576 ± 0.099[c]
1086	1.796	Ethyl (E)-4-heptenoate	54340-70-4	1376	1382 [101]	0.523 ± 0.054[a]	0.416 ± 0.053[a]	0.471 ± 0.039[a]
1122	1.925	Methyl octanoate	111-11-5	1390	1394 [104]	12.051 ± 0.400[a]	6.654 ± 0.318[b]	8.674 ± 0.609[c]
1214	0.868	Ethyl 2-hydroxyisovalerate	2441-06-7	1425	1427 [105]	0.991 ± 0.068[a]	0.955 ± 0.084[a]	0.970 ± 0.103[a]
1234	2.346	Ethyl octanoate	106-32-1	1439	1434 [71]	874.256 ± 77.852[a]	495.923 ± 36.067[b]	473.477 ± 39.811[c]
1286	2.737	Isopentyl hexanoate	2198-61-0	1456	1464 [87]	9.345 ± 0.474[a]	8.160 ± 0.578[b]	9.467 ± 0.713[a]
1498	0.871	Ethyl 2-hydroxy-4-methylvalerate	10348-47-7	1536	1515 [70]	13.102 ± 2.736[a]	12.231 ± 2.080[a]	10.727 ± 2.500[a]
1510	2.902	Butyl caprylate	589-75-3	1553	1601 [102]	1.452 ± 0.169[a]	0.952 ± 0.098[b]	1.439 ± 0.187[a]
1518	2.234	4-tert-Butylcyclohexyl acetate	32210-23-4	1696	--	1.545 ± 0.288[a]	1.948 ± 0.512[a]	1.629 ± 0.267[a]
1558	0.863	Isoamyl lactate	19329-89-6	1573	1570 [71]	11.817 ± 1.305[a]	10.963 ± 1.812[a]	10.875 ± 0.358[a]
1598	0.802	Diethyl malonate	105-53-3	1590	1580 [70]	0.819 ± 0.106[a]	0.842 ± 0.047[a]	0.790 ± 0.056[a]

1722	0.800	Ethyl methyl succinate	627-73-6	1644	1631 [106]	5.232 ± 0.532[a]	5.510 ± 0.382[a]	5.487 ± 0.442[a]
1730	1.101	Diethyl methylsuccinate	4676-51-1	1650	--	0.475 ± 0.053[a]	0.520 ± 0.048[a]	0.516 ± 0.037[a]
1762	0.983	Ethyl benzoate	93-89-0	1662	1644 [107]	1.466 ± 0.151[a]	1.195 ± 0.111[b]	1.011 ± 0.129[c]
1910	0.828	Benzyl acetate	140-11-4	1730	1726 [70]	0.068 ± 0.005[a]	0.064 ± 0.007[ab]	0.059 ± 0.004[b]
1942	0.509	Trimethylene acetate	628-66-0	1744	1660 [73]	0.688 ± 0.060[a]	0.585 ± 0.062[b]	0.567 ± 0.078[b]
1994	0.852	Methyl salicylate	119-36-8	1769	1778 [75]	0.403 ± 0.038[a]	0.369 ± 0.030[a]	0.371 ± 0.060[a]
2030	1.007	Diethyl glutarate	818-38-2	1784	1780 [70]	3.267 ± 0.299[a]	2.535 ± 0.290[b]	2.378 ± 0.121[b]
2034	0.921	Ethyl 2-phenylacetate	101-97-3	1785	1783 [70]	17.342 ± 1.450[a]	12.327 ± 2.450[b]	12.487 ± 1.122[b]
2066	0.976	Ethyl o-hydroxybenzoate	118-61-6	1802	1828 [94]	0.094 ± 0.009[a]	0.081 ± 0.008[a]	0.082 ± 0.011[a]
2090	0.900	2-Phenylethyl acetate	103-45-7	1814	1820 [73]	15.232 ± 1.246[a]	14.795 ± 0.804[a]	15.223 ± 0.323[a]
2230	1.024	Ethyl 3-phenylpropanoate	2021-28-5	1881	1879 [70]	0.733 ± 0.084[a]	0.547 ± 0.060[b]	0.548 ± 0.036[b]
2558	0.660	Diethyl hydroxybutanoate	626-11-9	2073	2062 [108]	1.091 ± 0.108[a]	0.764 ± 0.143[b]	0.739 ± 0.123[b]
2702	0.607	Ethyl 3-phenyl-2-propenoate	103-36-6	2137	2108 [107]	0.037 ± 0.003[a]	0.029 ± 0.003[b]	0.031 ± 0.004[ab]
		Ethers						
762	0.811	2-Furfuryl methyl ether	13679-46-4	1250	1247 [109]	0.303 ± 0.030[a]	0.271 ± 0.022[a]	0.262 ± 0.026[a]
1002	0.897	Anisole	100-66-3	1343	1341 [94]	0.170 ± 0.015[a]	0.138 ± 0.041[ab]	0.150 ± 0.004[b]
1090	0.648	3-Ethoxy-1-propanol	111-35-3	1377	1376 [73]	1.517 ± 0.102[a]	1.189 ± 0.151[b]	1.181 ± 0.155[b]
1238	1.210	Benzyl ether	103-50-4	2205	--	1.869 ± 0.130[a]	1.687 ± 0.062[b]	1.651 ± 0.093[b]
		Furan derivatives						
158	0.657	Tetrahydrofuran	109-99-9	873	857 [110]	0.155 ± 0.015[a]	0.114 ± 0.026[b]	0.093 ± 0.016[b]
1322	0.559	Furfural	98-01-1	1472	1460 [73]	42.629 ± 3.157[a]	29.965 ± 0.761[b]	27.073 ± 1.046[c]
1394	0.811	Benzofuran	271-89-6	1500	1489 [111]	1.774 ± 0.091[a]	1.142 ± 0.130[b]	1.228 ± 0.098[b]
1410	0.625	1-(2-Furanyl)-ethanone	1192-62-7	1507	1510 [109]	2.716 ± 0.055[a]	2.732 ± 0.125[a]	2.558 ± 0.049[b]

1570	0.641	5-Methyl-2-furfural	620-02-0	1578	1578 [109]	7.186 ± 1.223[a]	4.216 ± 0.656[b]	4.355 ± 0.545[b]
1598	0.958	2-Methylbenzofuran	4265-25-2	1590	1563 [112]	0.517 ± 0.041[a]	0.338 ± 0.046[b]	0.425 ± 0.032[c]
1658	0.721	2-Acetyl-5-methylfuran	1193-79-9	1616	1608 [113]	0.027 ± 0.005[a]	0.031 ± 0.008[a]	0.021 ± 0.009[a]
1686	0.729	Ethyl 2-furoate	614-99-3	1628	1621 [71]	7.447 ± 0.205[a]	7.248 ± 0.172[a]	7.289 ± 0.313[a]
1782	0.470	2-Furanmethanol	98-00-0	1671	1669 [114]	14.455 ± 1.163[a]	13.236 ± 0.522[a]	13.724 ± 0.449[a]
1866	0.517	Dihydro-3-methylene-2,5-furandione	2170-03-8	1682	1680 [115]	0.162 ± 0.011[a]	0.104 ± 0.018[b]	0.116 ± 0.011[b]
1962	0.481	2(5H)-Furanone	497-23-4	1754	1767 [116]	0.237 ± 0.043[a]	0.156 ± 0.013[b]	0.150 ± 0.025[b]
		Ketones						
378	0.710	2,3-Pentanedione	600-14-6	1064	1068 [65]	0.351 ± 0.034[a]	0.237 ± 0.043[b]	0.243 ± 0.035[b]
610	1.335	2-Heptanone	110-43-0	1186	1183 [117]	0.314 ± 0.028[a]	0.324 ± 0.034[a]	0.264 ± 0.036[b]
782	1.602	3-Octanone	106-68-3	1257	1261 [84]	0.349 ± 0.041[a]	0.319 ± 0.026[ab]	0.284 ± 0.028[b]
910	1.685	2,2,6-Trimethylcyclohexanone	2408-37-9	1313	1312 [118]	0.496 ± 0.016[a]	0.480 ± 0.016[b]	0.473 ± 0.017[b]
1118	1.652	2-Nonanone	821-55-6	1387	1387 [71]	0.942 ± 0.046[a]	0.909 ± 0.034[a]	0.643 ± 0.124[b]
1486	1.619	3-Hexen-2-one	763-93-9	1555	--	0.013 ± 0.001[a]	0.012 ± 0.001[a]	0.012 ± 0.001[a]
1594	0.533	2-Cyclopentene-1,4-dione	930-60-9	1588	1576 [119]	0.642 ± 0.148[a]	0.533 ± 0.154[ab]	0.443 ± 0.091[b]
1618	1.839	2-Undecanone	112-12-9	1597	1599 [120]	0.293 ± 0.040[a]	0.191 ± 0.014[b]	0.217 ± 0.040[b]
		Lactones						
1834	0.666	γ-Caprolactone	695-06-7	1695	1694 [72]	0.092 ± 0.019[a]	0.089 ± 0.005[a]	0.079 ± 0.010[a]
2478	0.572	Dehydromevalonic lactone	2381-87-5	1953	--	0.110 ± 0.021[a]	0.093 ± 0.020[a]	0.058 ± 0.010[b]
2498	0.827	γ-Nonalactone	104-61-0	1958	1998 [103]	0.138 ± 0.016[a]	0.151 ± 0.010[a]	0.152 ± 0.010[a]
2522	0.472	Pantolactone	599-04-2	1994	2033 [121]	0.409 ± 0.036[a]	0.359 ± 0.049[a]	0.326 ± 0.105[a]
		Naphthalene compounds						
1906	0.919	Naphthalene	91-20-3	1728	1740 [92]	0.182 ± 0.019[a]	0.173 ± 0.022[a]	0.114 ± 0.009[b]

2138	1.005	1-Methylnaphthalene	90-12-0	1869	1875 [122]	0.058 ± 0.016[a]	0.048 ± 0.015[b]	0.047 ± 0.005[b]
2430	1.069	1,8-Dimethylnaphthalene	569-41-5	1959	1989 [123]	0.089 ± 0.016[a]	0.053 ± 0.009[b]	0.079 ± 0.029[a]
		Norisoprenoids						
1702	1.178	Safranal	116-26-7	1636	--	0.242 ± 0.010[a]	0.229 ± 0.010[ab]	0.219 ± 0.014[b]
1958	1.501	β-Damascenone	23726-93-4	1811	1830 [65]	1.399 ± 0.096[a]	1.165 ± 0.031[b]	0.841 ± 0.054[c]
2002	0.895	Tetrahydroionol	4361-23-3	1790	--	1.391 ± 0.122[a]	1.161 ± 0.058[b]	1.181 ± 0.027[b]
		Phenols						
2190	0.577	Guaiacol	90-05-1	1862	1867 [65]	0.675 ± 0.099[a]	0.489 ± 0.079[b]	0.470 ± 0.069[a]
2382	0.625	Creosol	93-51-6	1929	1956 [93]	0.094 ± 0.005[a]	0.109 ± 0.009[b]	0.106 ± 0.009[ab]
2486	0.438	Phenol	108-95-2	1979	2008 [65]	0.400 ± 0.038[a]	0.289 ± 0.041[b]	0.395 ± 0.073[a]
2486	0.477	2-Methylphenol	95-48-7	1975	1969 [124]	0.115 ± 0.005[a]	0.106 ± 0.015[ab]	0.094 ± 0.011[b]
2522	0.693	4-Ethylguaiacol	2785-89-9	1994	2002 [125]	1.931 ± 0.114[a]	2.006 ± 0.094[a]	1.813 ± 0.177[a]
2678	0.557	2-Methoxy-4-propylphenol	2785-87-7	2035	2081 [124]	0.098 ± 0.014[a]	0.099 ± 0.011[a]	0.117 ± 0.010[b]
2846	0.468	2,4-Di-tert-butylphenol	96-76-4	2196	2280 [119]	0.561 ± 0.091[a]	0.365 ± 0.053[b]	0.326 ± 0.052[b]
		Sulphur compounds						
350	0.742	Methylthioacetate	1534-08-3	1066	1057 [126]	0.362 ± 0.038[a]	0.370 ± 0.025[a]	0.628 ± 0.052[b]
382	0.858	Dimethyl disulfide	624-92-0	1067	1063 [127]	0.183 ± 0.024[a]	0.248 ± 0.043[b]	0.323 ± 0.033[c]
1442	0.768	2-Methyl-3-thiolanone	13679-85-1	1520	1525 [119]	0.579 ± 0.047[a]	0.597 ± 0.086[a]	0.810 ± 0.050[b]
1834	0.595	2-Thiophenecarboxaldehyde	98-03-3	1694	1684 [70]	0.186 ± 0.006[a]	0.179 ± 0.007[a]	0.263 ± 0.008[b]
1886	0.549	3-(Methylthio)-1-propanol	505-10-2	1719	1719 [73]	5.452 ± 0.345[a]	6.404 ± 0.903[a]	8.198 ± 0.816[b]
		Terpenic compounds						
434	2.715	Linalool-3,7-oxide	7392-19-0	1117	1112 [122]	0.794 ± 0.059[a]	0.791 ± 0.090[a]	0.789 ± 0.069[a]
518	2.785	β-Myrcene	123-35-3	1157	1161 [128]	0.283 ± 0.056[a]	0.218 ± 0.053[a]	0.226 ± 0.059[a]

618	2.976	Limonene	5989-54-8	1190	1191 [128]	5.986 ± 0.384[a]	1.472 ± 0.275[b]	1.569 ± 0.321[b]
638	2.780	Eucalyptol	470-82-6	1198	1195 [128]	1.163 ± 0.135[a]	0.304 ± 0.047[b]	0.229 ± 0.025[c]
730	2.538	trans-β-Ocimene	3779-61-1	1247	1250 [129]	0.313 ± 0.048[a]	0.265 ± 0.052[b]	0.257 ± 0.076[b]
742	2.792	γ-Terpinene	99-85-4	1249	1274 [79]	0.366 ± 0.046[a]	0.254 ± 0.068[b]	0.339 ± 0.042[a]
786	2.617	*p*-Cymene	99-87-6	1264	1265 [130]	0.027 ± 0.005[a]	0.044 ± 0.015[a]	0.029 ± 0.005[a]
830	2.786	α-Terpinolene	586-62-9	1283	1315 [79]	1.051 ± 0.034[a]	0.210 ± 0.022[b]	0.357 ± 0.030[c]
1014	2.126	Rose oxide	16409-43-1	1348	1339 [99]	0.406 ± 0.033[a]	0.228 ± 0.016[b]	0.234 ± 0.020[b]
1310	1.681	Nerol oxide	1786-08-9	1465	1469 [105]	5.762 ± 0.414[a]	4.736 ± 0.265[b]	4.727 ± 0.228[b]
1382	1.432	Camphor	76-22-2	1475	1458 [121]	0.283 ± 0.044	ND	ND
1398	2.030	Bornylene	464-17-5	1512	--	0.724 ± 0.053[a]	0.738 ± 0.067[a]	0.730 ± 0.048[a]
1514	0.983	Linalool	78-70-6	1555	1526 [128]	7.993 ± 0.783[a]	6.966 ± 0.429[a]	5.920 ± 0.439[b]
1618	1.188	Terpinen-4-ol	562-74-3	1598	1605 [107]	1.105 ± 0.131[a]	0.971 ± 0.074[ab]	0.913 ± 0.043[b]
1658	0.870	Hotrienol	29957-43-5	1616	1613 [131]	0.439 ± 0.053[a]	0.581 ± 0.041[b]	0.560 ± 0.038[b]
1838	0.962	α-Terpineol	98-55-5	1696	1686 [132]	8.094 ± 0.364[a]	6.022 ± 0.521[b]	4.149 ± 0.202[c]
1870	3.085	α-Muurolene	10208-80-7	1713	1707 [133]	0.436 ± 0.051[a]	0.270 ± 0.033[b]	0.272 ± 0.056[b]
1890	3.080	β- Bisabolene	495-61-4	1720	1719 [134]	0.181 ± 0.019[a]	0.151 ± 0.012[b]	0.136 ± 0.023[b]
1898	2.957	α-Caryophyllene	6753-98-6	1724	1705 [135]	0.110 ± 0.016[a]	0.076 ± 0.013[b]	0.072 ± 0.016[b]
2174	0.818	Nerol	106-25-2	1854	1803 [136]	0.536 ± 0.039[a]	0.407 ± 0.042[b]	0.318 ± 0.041[c]
2184	0.815	Geraniol	106-24-1	1874	1867 [79]	0.495 ± 0.042[a]	0.361 ± 0.038[b]	0.439 ± 0.020[a]
2189	2.345	Spathulenol	6750-60-3	2059	2106 [137]	0.090 ± 0.014[a]	0.076 ± 0.013[a]	0.077 ± 0.021[a]
2494	1.360	Cedrol	77-53-2	2056	2069 [138]	0.097 ± 0.014[a]	0.084 ± 0.010[a]	0.118 ± 0.010[a]
2550	1.454	Nerolidol	7212-44-4	1971	1991 [139]	0.663 ± 0.089[a]	0.417 ± 0.063[b]	0.499 ± 0.027[c]
2782	0.646	Cadelene	483-78-3	2211	2200 [98]	0.264 ± 0.020[a]	0.159 ± 0.019[b]	0.197 ± 0.049[b]

		Other Compounds						
1042	1.411	Indane	496-11-7	1365	1365 [92]	0.050 ± 0.008[a]	0.042 ± 0.005[a]	0.063 ± 0.011[b]
1410	1.117	Ethyl sorbate	2396-84-1	1511	1501 [93]	0.052 ± 0.008[a]	0.053 ± 0.004[a]	0.055 ± 0.010[a]
1622	0.656	Benzonitrile	100-47-0	1600	1583 [140]	1.272 ± 0.164[a]	1.410 ± 0.391[a]	1.464 ± 0.230[a]
1634	0.773	1-Ethyl-2-formylpyrrole	2167-14-8	1605	1610 [141]	0.479 ± 0.036[a]	0.564 ± 0.039[b]	0.597 ± 0.042[b]
1666	0.674	1-Methyl-2-formylpyrrole	1192-58-1	1619	1620 [92]	0.148 ± 0.015[a]	0.182 ± 0.042[ab]	0.193 ± 0.031[b]
2050	0.586	Tetrahydro-2H-pyran-2-one	542-28-9	1794	1780 [107]	0.188 ± 0.033[a]	0.221 ± 0.039[a]	0.207 ± 0.050[a]

[#] Retention time for first (^1t_R) and second (^2t_R) dimensions in seconds. [$] RI_{Calc}: Retention index obtained through the modulated chromatogram. [*] RI_{Lit}: Retention index reported in the literature for the DB-FFAP column or equivalents. [&]The results are expressed as the averages of 3 bottles × 3 replicates ($n = 9$) ± the standard deviation. The compounds were quantified, and their concentration was expressed as equivalents of 3-octanol (μg/L). Different superscript lowercase letters in a row represent statistically significant differences between wines bottled with different stoppers at $p < 0.05$, using Two-Way ANOVA (followed by Tukey´s multiple comparison test) in GraphPad Prism. ND—not detected.
